# Turning the FeFe hydrogenase from *Clostridium beijerinckii* into an efficient H_2_ oxidation catalyst using a redox-active matrix

**DOI:** 10.1073/pnas.2514698122

**Published:** 2025-10-09

**Authors:** Dawit T. Filmon, Jan Jaenecke, Martin Winkler, Vincent Fourmond, Christophe Léger, Nicolas Plumeré

**Affiliations:** ^a^Technical University of Munich (TUM), Campus Straubing for Biotechnology and Sustainability, Uferstraße 53, Straubing 94315, Germany; ^b^CNRS, Aix-Marseille Universite, Laboratoire de Bioénergétique et Ingénierie des Protéines, Marseille 13009, France

**Keywords:** hydrogenase, oxygen resistance, hydrogen oxidation, redox-active films, catalyst reactivation

## Abstract

Hydrogenases are Nature’s sustainable and efficient catalysts for the conversion between H^+^ and H_2_. The obstacles that prevented their use as H_2_ oxidation catalysts in fuel cells are being removed one by one, thanks to the continuous discovery of hydrogenases that have unexpected catalytic properties, the development of new methods for their scalable production and matrices that protect them. Obtaining an efficient biohybrid electrode that is scalable and robust under a large range of experimental conditions is still challenging. The FeFe hydrogenase of *Clostridium beijerinckii* is a very active catalyst of H_2_ evolution and can be handled under O_2_, and its production can potentially be scaled up. However, it was believed that it cannot be used for H_2_ oxidation, as it is easily oxidized to an O_2_-stable but inactive state. Here, we show that when the enzyme is embedded into a redox-active film whose reduction potential is finely tuned to the equilibrium potential of the H^+^/H_2_ couple, the potential that the enzyme experiences can be buffered in a sharp window that is actually compatible with enzyme-catalyzed H_2_ oxidation and prevents anaerobic inactivation. This leads us to provide the demonstration of an FeFe hydrogenase-based system that can be used for H_2_ oxidation and that can be repeatedly exposed to O_2_ during and between operational cycles.

## Significance

Hydrogenases are large and efficient metalloenzymes that catalyze the conversion between protons and hydrogen, with an inorganic active site that coordinates cheap transition metals. They have long been considered to replace precious metals as catalysts in fuel cells, but despite their diversity in the microbial world, none of the hydrogenases identified so far meet all the requirements. The FeFe hydrogenase from the bacterium *Clostridium beijerinckii* comes close: Its production can be scaled up and it can be exposed to air without being destroyed. However, its protection from oxidative damage also prevents H_2_ oxidation catalysis. We show that the enzyme can actually oxidize H_2_, even in the presence of O_2_, when it is embedded in a finely tuned redox environment.

The use of hydrogenases as alternatives to precious metal catalysts (1) for H_2_ oxidation in fuel cells has been a very active field of research (2–6). The main challenge in using hydrogenases comes from their sensitivity to oxidative (anaerobic or aerobic) conditions (1). There are various ways around this issue. One is to embed the fragile enzyme into a protective matrix (7, 8). The other is to explore the biodiversity to identify, produce, characterize, and eventually use enzymes that are particularly robust (9).

Two main families of hydrogenases have been considered, NiFe and FeFe hydrogenases (the names of these enzymes refer to the metal content of their active site). Some hydrogenases are described as “O_2_-sensitive” because they quickly react with O_2_ to produce inactive states. This inactivation may be irreversible (the active site is destroyed, as occurs with most FeFe hydrogenases) or reversible. If the activity is recovered upon reduction, the enzyme is referred to as “O_2_-stable”; this is the case of all standard NiFe hydrogenases and the FeFe hydrogenase from *Clostridium beijerinckii*. If this reactivation is fast even above the H^+^/H_2_ equilibrium potential, the enzyme can oxidize H_2_ in the presence of O_2_ and is called “O_2_-resistant.” This is the case of the NiFe hydrogenases from the phylogenetic group 1d (9, 10).

Because the presence of O_2_ is unavoidable in H_2_/O_2_ fuel cells, early efforts focused on using these O_2_-resistant NiFe hydrogenases (11–14). The subclass of NiFeSe hydrogenases has also been considered because these O_2_-stable enzymes reactivate relatively quickly upon reduction (15, 16). However, the biosynthesis of NiFe hydrogenases involves a very heavy biological machinery, and despite recent advances (17), they cannot be produced in large quantities, limiting their potential as a scalable alternative to precious metal catalysts.

**Fig. 1.**
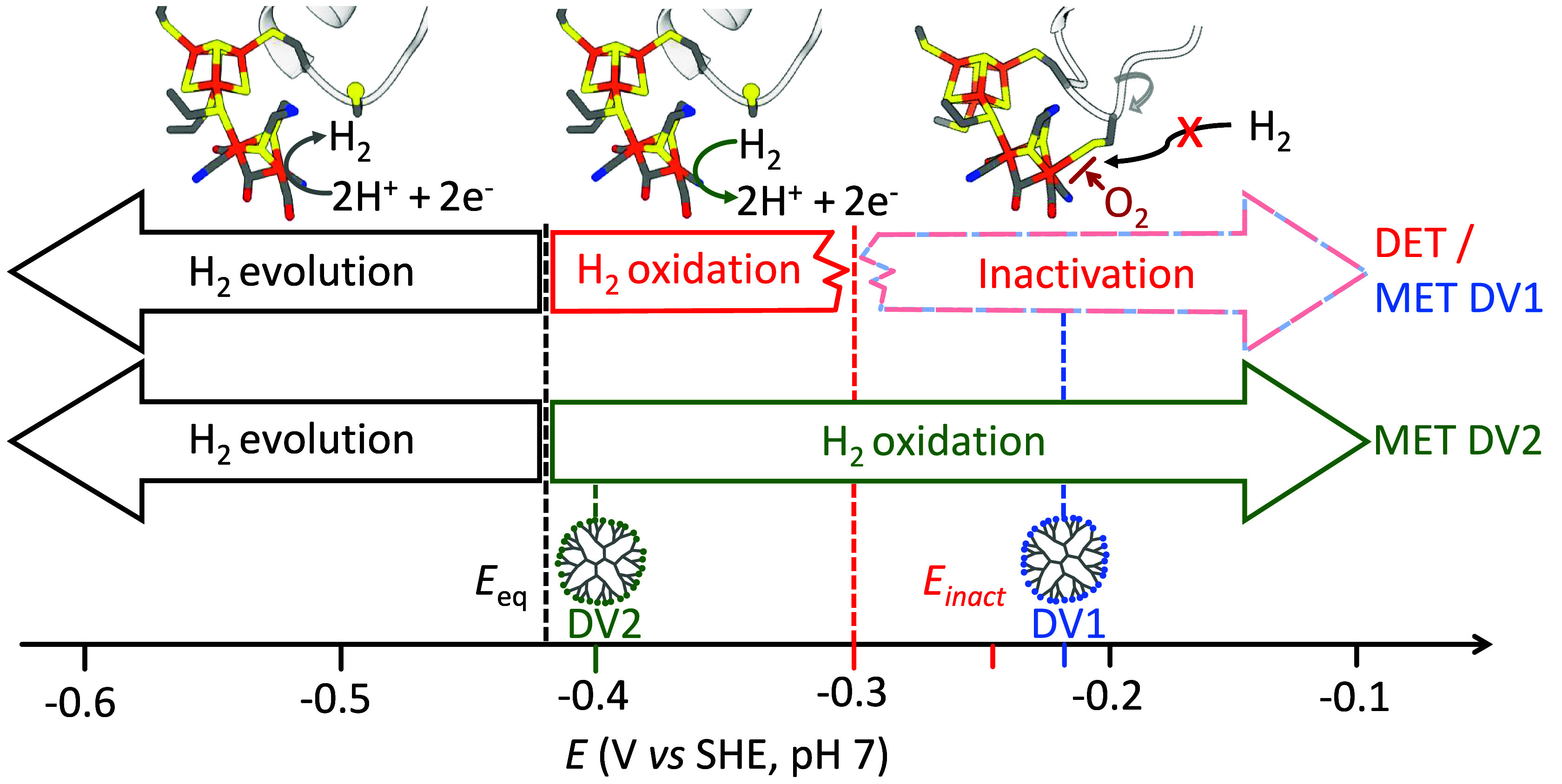
Potential window for H_2_ evolution, H_2_ oxidation, and inactivation of the FeFe hydrogenase from **C.* bejerinckii* (CbA5H) under conditions of DET with electrodes and for the same enzyme when embedded in redox-active films made of dendrimers DV1 and DV2 (mediated electron transfer, MET). Under DET, the catalytic current for H_2_ oxidation is lost at potentials more positive than −0.3 V vs. Standard Hydrogen Electrode (SHE) (red dashed line). DV1 fails to support H_2_ oxidation because its redox potential lies above the potential where the enzyme inactivates. In contrast, DV2 has a redox potential that aligns with the narrow potential window in which CbA5H remains active for H_2_ oxidation. The redox buffering effect of DV2 maintains the hydrogenase in its active form regardless of the applied potential at the electrode, which extends its catalytic activity for H_2_ oxidation far beyond *E*_inact_.

In contrast, recent progress in the artificial maturation of FeFe hydrogenases (18), whose active site is a dinuclear cluster of Fe (Fig. 1), facilitated the production of some of them by heterologous production of the apo-enzymes (devoid of dinuclear cluster) and their activation upon subsequent incorporation (19, 20) of a synthetically produced (21) diiron complex. All steps are potentially compatible with a large-scale production of the enzyme. However, FeFe hydrogenases are deemed very O_2_-sensitive: none discovered so far can oxidize H_2_ in the presence of O_2_ under conditions of direct electron transfer (DET), and in the case of most FeFe hydrogenase characterized so far, the binding of O_2_ to their active site leads to irreversible degradation in a multistep process (22–24).

The use of these O_2_-sensitive FeFe hydrogenases is enabled by embedding them in redox-active matrices for protection from O_2_ under aerobic turnover conditions for H_2_ oxidation (7). However, this protection from O_2_ is strictly dependent on the presence of H_2_, and thus it is effective only under operational conditions, but not during shutdown or storage. Some FeFe hydrogenases can be protected via inhibition by exogenous sulfide (25, 26) because sulfide and O_2_ compete for binding to the same coordination site (Fig. 1*B*) but this is only useful for electrode preparation and handling before catalysis. The redox-driven release of the sulfide ligand activates the enzyme and makes it O_2_ sensitive (27, 28).

The recently discovered FeFe hydrogenase from **C.* bejerinckii* (CbA5H) is unusual because the sulfide ligand, whose binding to the active site Fe ion can prevent O_2_ attack, is intrinsically present as a cysteine side chain borne by a flexible loop (Fig. 1*C*) (19, 29–31). The Fe–cysteine bond is tight under oxidative conditions, e.g., in the presence of O_2_ or when the enzyme is wired to an electrode whose potential is moderately high (> −0.3 V vs. SHE, pH 7.0). Based on the data in ref. 29, we estimate an inactivation potential *E*_inact_ ≃ −250 mV vs. SHE at pH 7 (or +170 mV vs. RHE) from the value of the electrode potential where the inactivation and reactivation rate constants are equal. The distal Fe is also the H_2_ binding site, and the oxidation (upon exposure to air or mildly oxidative potentials in DET experiments) that triggers this protection also makes the enzyme inactive. This restricts the potential range for enzyme-catalyzed H_2_ oxidation to a narrow window, strongly limiting its catalytic performance and initially suggesting that the enzyme was unsuitable for H_2_ oxidation (19, 29–31).

Here, we show that the O_2_-stable FeFe hydrogenase CbA5H can actually sustain H_2_ oxidation under very oxidizing conditions, provided that it is embedded into a redox-active film, but only on condition that the redox properties of the latter are finely tuned to the H^+^/H_2_ equilibrium potential (*E*_eq_). The polymer matrix also makes the system O_2_-resistant. We could therefore design a hybrid H_2_ oxidizing electrode based on a scalable FeFe hydrogenase that remains active under all conditions, both aerobic and anaerobic, both in the presence and in the absence of H_2_.

## Results

**Fig. 2.**
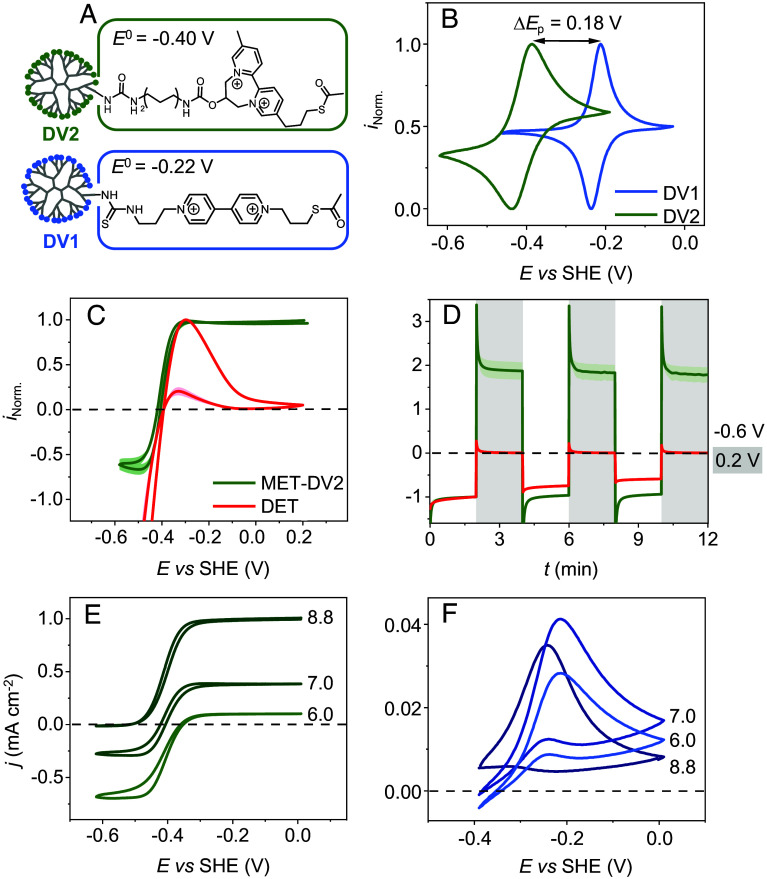
Prevention of high-potential inactivation of CbA5H via MET. (*A*) Chemical structure of the viologen moieties in the dendrimers DV1 and DV2. (*B*) CVs of glassy carbon electrodes modified with DV1 (blue) and DV2 (green) at pH 7.0. DV1 (2.8 µg) and DV2 (0.57 µg) were deposited by drop-casting on the electrodes. The currents are normalized by the maximal positive current. (*C*) CVs of CbA5H under DET conditions (red) and embedded in redox hydrogel films of DV2 (green) at pH 7.0. The current is normalized by the maximal positive current. (*D*) Chronoamperometry (CA) of CbA5H under DET conditions (red) and embedded in films of DV2 (green) at −0.6 and +0.2 V vs. SHE at pH 7.0. The catalytic current is normalized by the reductive current measured at *t* = 2 min. CVs of CbA5H (0.16 nmol) embedded in films of DV2 (0.57 µg) (*E*) or of DV1 (2.8 µg) (*F*) at pH 8.8, 7.0, and 6.0. All MET measurements were conducted on a glassy carbon electrode (*d* = 3 mm) in Tris-HCl/Citrate buffer (50 mM) with KCl (100 mM). All DET measurements were conducted on a pyrolytic graphite edge electrode (4 × 4 mm^2^) in phosphate buffer (100 mM) with NaCl (100 mM). All experiments were conducted at *v* = 10 mV s^−1^, T = 20 °C, ω = 2,000 rpm, 100% H_2_ atmosphere. The nonnormalized CVs of *C* and *D* are shown in *SI Appendix*, Figs. S1 and S2. The SD of *E* and *F* are shown in *SI Appendix*, Figs. S3 and S4, respectively. Limitations of H_2_ mass transport were excluded via control experiments showing that the catalytic current is independent of the rotation rate at high electrode rotation rates (*SI Appendix*, Fig. S5), and of H_2_ concentration at high H_2_ concentrations (*SI Appendix*, Fig. S6). CVs of CbA5H embedded in DV2 with different amounts show a linear increase in catalytic current, which is consistent with the behavior of thin films (*SI Appendix*, Fig. S7) (34).

We used previously reported viologen-functionalized dendrimers to immobilize hydrogenases within redox-active films on electrode surfaces. Although polymeric backbones are more commonly used for hydrogenase immobilization, we chose the dendrimeric backbones for their ability to form thin, homogeneous films, which facilitate the quantitative analysis of the resulting current response (32, 33). The viologen-modified dendrimers (Fig. 2*A*) were based on either alkylated 4,4′-bipyridine (DV1) or on alkylated 2,2′-bipyridine (DV2) bearing thioacetate groups to enable crosslinking for film formation (32, 34). The redox potentials of 2,2′-viologens are generally more negative than those of 4,4′-viologens, due to ring-induced strain that destabilizes the radical cation formed upon one-electron reduction (35). We used cyclic voltammetry of films based on DV1 and DV2 dendrimers to determine their redox potentials, yielding values of −0.22 and −0.40 V vs. SHE, respectively (Fig. 2*B*). In this figure, the cyclic voltammogram (CV) of DV1 exhibits characteristics intermediate between diffusion-controlled and surface-confined behavior (36), whereas the CV of DV2 displays a more pronounced diffusion-like behavior, indicating differences in film thicknesses between the two systems.

We recorded CVs for the hydrogenase from CbA5H under two different electron transfer conditions: DET (Fig. 2*C*, red), achieved by simple enzyme adsorption onto the electrode, and mediated electron transfer (MET, Fig. 2*C*, green), where the enzyme is embedded in a film of DV2. Both measurements were conducted with the electrodes rotating in a solution saturated with H_2_, under anaerobic conditions. In both DET and MET cases, H_2_ evolution is observed as a negative current below the equilibrium potential (*E*_eq_ = −0.42 V vs. SHE at pH 7), and H_2_ oxidation occurs above this value. However, under DET conditions, the enzyme inactivates at potentials above −0.3 V as indicated by a drop in H_2_ oxidation current as reported by several groups (19, 30, 37). The subsequent recovery of the reductive current in the return sweep to low potential shows that this inactivation is reversible. It is attributed to the binding of the cysteine to the active site (Fig. 1) under mildly oxidizing conditions (29). In contrast, under conditions of MET, the H_2_ oxidation current is stable even at potentials up to 0.2 V vs. SHE (green in Fig. 2*C*). The observed plateau current across the potential range between −0.3 and 0.2 V vs. SHE is characteristic of catalysis by redox enzymes embedded in redox-active films on rotating electrodes, operating under conditions that are free from inactivation (34, 38). This difference in response to oxidative potential is further confirmed by chronoamperometry (CA) experiments (Fig. 2*D*), where the potential is stepped between −0.6 V and +0.2 V vs. SHE: Under MET conditions, both H_2_ oxidation and evolution are observed (green in Fig. 2*D*), while under DET conditions, only H_2_ evolution is visible (red in Fig. 2*D*).

To determine whether high-potential-induced inactivation can also occur under conditions of MET, we repeated the MET experiment using the more oxidizing 4,4’-viologen modified redox dendrimer (DV1, Fig. 2*F*) whose potential is 0.18 V more positive than that of DV2 (*E*^0^ = −0.22 V vs. SHE, Fig. 2*B*). Fig. 2 *E* and *F* compare CVs of Cb5AH embedded into films of DV2 (panel *E*) and DV1 (panel *F*) at various pH values. In the case of DV2, the pH values of 6, 7, and 8.8 correspond to relatively small *E*^0^ – *E*(H^+^/H_2_) values (–40, 20, and 120 mV, respectively), and the CVs show sustained H_2_ oxidation without signs of inactivation. The catalytic bias shifts toward H_2_ oxidation with increasing pH as described previously (34) (Fig. 2*E*). In contrast, for DV1, the corresponding *E*^0^ – *E*(H^+^/H_2_) values are much higher (140, 200, and 300 mV), and all CVs exhibit anaerobic inactivation (Fig. 2*F*). At the higher pH values, the inactivation becomes more pronounced as indicated by the steeper slope in the current decrease at higher potentials (Fig. 2*F*). This is attributed to the *E*^0^ of the viologen being pH independent, whereas the kinetics of inactivation is pH dependent (29). At neutral and acidic pHs, the inactivation is slightly less pronounced, however, the catalysis is shifted toward H_2_ uptake due to the mismatch of the mediator potential and catalysis potential (34).

**Fig. 3.**
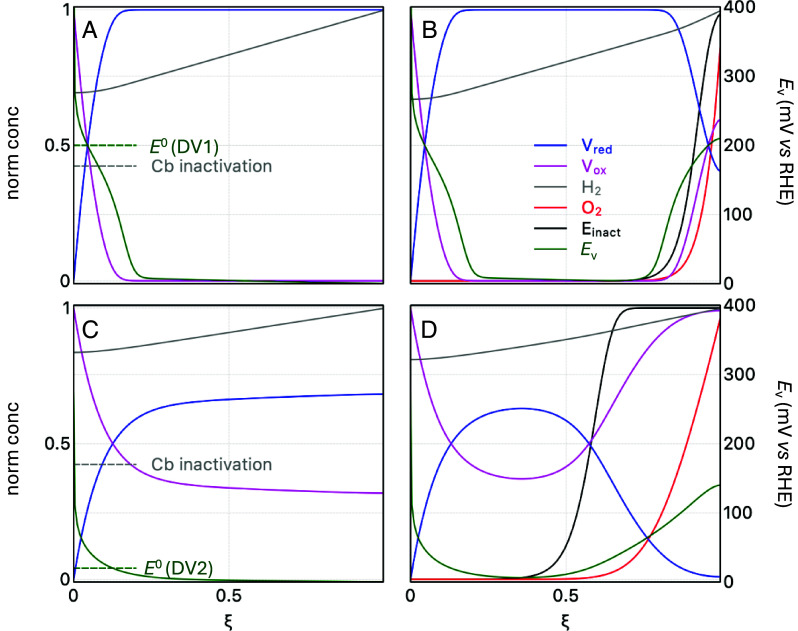
Simulation of the distribution of the different species within the films in the presence and absence of O_2_. Concentration profiles of V_ox_ (pink), V_red_ (blue), H_2_ (gray), O_2_ (red), and inactive enzyme (black), and the viologen Nernst potential vs. Reversible Hydrogen Electrode (RHE) (dark green and *Right Y*-axis) (calculated using the Nernst equation from the ratio V_ox_/V_red_) as a function of the normalized distance from the electrode, with viologen potentials matching those of DV1 (Top, panels A and B) and DV2 (Bottom, panels C and D), calculated under oxidizing, anaerobic conditions (*Left*, panels *A* and *C*) and aerobic conditions (*Right*, panels *B* and *D*). The anaerobic concentration profiles are in the steady-state, the aerobic concentration profiles are snapshots calculated at the same time after exposure to O_2_. The standard potentials of the viologens and the potential where the enzyme inactivates (29) are constant parameters and shown as horizontal dashed lines (they do not depend on distance, unlike the Nernst potential). See SI for details about the simulations.

To better understand the effect of changing the potential of the viologen (*E*^0^), we calculated its Nernst potential (*E*_V_) within the redox-active films using the concentration profiles of oxidized and reduced viologen, which were obtained by solving a kinetic reaction–diffusion model. Unlike in our previous work (39–41), this model assumes bidirectional H^+^/H_2_ conversion by the enzyme (see details in *SI Appendix*). The simulations were performed using parameters relevant to the experiments carried out at pH 6, 7, and 8.8 (shown in Fig. 2 *E* and *F*), considering the *E*^0^ values of DV1 and DV2 relative to the H^+^/H_2_ equilibrium potential, keeping all other parameters constant. The results of the simulation for pH 7 are shown in Fig. 3, and those for pH 6 and 8.8 are shown in *SI Appendix*, Figs. S8 and S9, respectively.

Panels *A* and *C* show the steady-state anaerobic concentration profiles. The concentration profiles of H_2_ (gray), oxidized viologen (pink), and reduced viologen (blue), along with the Nernst potential of the viologen (dark green and right Y-axis), are shown as a function of the normalized distance from the electrode (ξ = 0 at the electrode surface to ξ = 1 at the film/solution interface), under H_2_ oxidation conditions (i.e., high electrode potential) and in the absence (left column) or presence (right column) of O_2_. In all cases, the simulations reveal a slight depletion of H_2_ within the film (the H_2_ concentration is lower than in the bulk of the solution), and the accumulation of oxidized viologen near the electrode surface. The Nernst potential of the viologen matches the electrode potential only in a tiny region close to the electrode. Away from the electrode (ξ < 0.2), where catalytic H_2_ oxidation occurs, it decreases to a value that equates roughly the *E*^0^ of the viologen (marked by horizontal dashed green lines), and further from the electrode (0.2 < ξ < 1), it equilibrates with that of the H^+^/H_2_ couple, irrespective of the standard potential of the viologen, so that *E*_V_ tends to 0 vs. RHE.

Fig. 3 also indicates with a horizontal dashed gray line the approximate value of the inactivation potential of CbA5H (≃0.17 V vs. RHE). The effect of using one redox-active dendrimer or the other is clear from Fig. 3: The Nernst potential that is experienced by the enzyme in the region close to the electrode where H_2_ oxidation occurs is higher or lower (DV1 and DV2, respectively) than the inactivation potential of CbA5H. This observation is therefore consistent with the inactivation not occurring when the enzyme is embedded in DV2 (*E*^0^ = –0.4 V vs. SHE, Fig. 2*E*).

The same is observed over a range of pH from 6 to 8.8 (see the simulations in panels *A* and *C* of *SI Appendix*, Figs. S8 and S9, respectively). The potential of DV2 remains below *E*_inact_ which explains the sustained H_2_ oxidation in this pH range (Fig. 2*E*). In contrast, DV1, is higher or near *E*_inact_ in this pH range, leading to deactivation (Fig. 2*F*).

Having shown that the hydrogenase from CbA5H can be protected from oxidative, anaerobic inactivation under conditions of MET, we turn to the protection from aerobic inactivation. To assess the ability of redox-active films to protect the enzyme from O_2_, we incorporated CbA5H into the DV2 dendrimer system and into a previously reported 2,2′-viologen-modified polyvinyl alcohol (PV2) matrix (34). PV2 bears the same viologen moiety as DV2 and displays a similar redox potential of −0.431 ± 0.007 V vs. SHE. PV2 was selected as an additional matrix for the aerobic experiments because it enables the formation of more stable films compared to DV2, possibly due to the larger molecular weight of its building blocks. This enhanced stability makes PV2 well-suited for studying the resistance of CbA5H films to O_2_ upon prolonged exposure. Under anaerobic conditions, CbA5H embedded in PV2 films show sustained currents for H_2_ oxidation (*SI Appendix*, Fig. S11) comparable to those observed with DV2 (Fig. 2*E*). This demonstrates that PV2, like DV2, effectively protects CbA5H from high-potential inactivation, which is expected since PV2 and DV2 have similar redox potentials.

### Stability in Air in the Absence of H_2_.

**Fig. 4.**
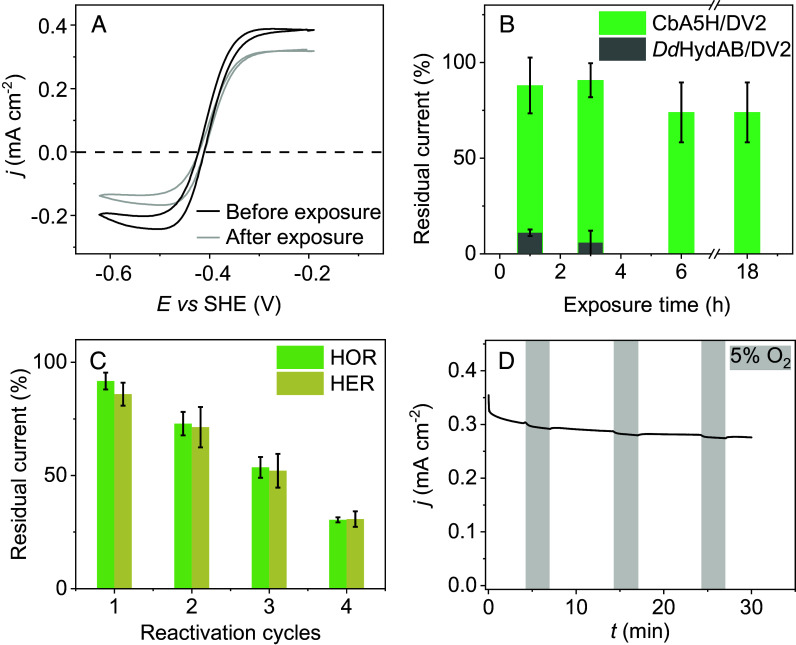
Prevention of O_2_-induced inactivation of CbA5H embedded in viologen-modified films. (*A*) CVs of CbA5H (0.16 nmol) embedded in DV2 (0.57 µg) films in Tris-HCl/Citrate buffer (50 mM) with KCl (100 mM) saturated with H_2_ (100%) at pH 7.0 before and after 3 h of exposure to air at 5 °C. (*B*) Residual current for H_2_ oxidation obtained from CVs of redox-active films containing either CbA5H or sulfide-protected DdHydAB after operating the electrode for H_2_ oxidation followed by exposure to air for different periods of time. The corresponding CVs of DdHydAB embedded in DV2 before and after the exposure to air are shown in *SI Appendix*, Fig. S10 [note that the protection of DdHydAB by sulfide is only operational before the film is used for catalysis (27, 28)]. (*C*) Residual H_2_ oxidation currents obtained from CVs of the CbA5H/DV2-modified electrode after repeated cycles of H_2_ oxidation followed by O_2_-exposure (at 5 °C for 1 h). The currents are normalized with the values obtained for the first CV before exposure to O_2_. (*D*) CAs of CbA5H (0.16 nmol) in PV2 (1.13 µg) at −0.2 V vs. SHE with a 95:5 gas feed of H_2_:N_2_ (nonshaded area) and H_2_:O_2_ (gray area). Unless specified otherwise, all experiments were conducted on a glassy carbon electrode (*d* = 3 mm), v = 10 mV s^−1^, T = 20 °C, ω = 2,000 rpm, 100% H_2_ atmosphere.

CVs of DV2/CbA5H-modified electrodes before (black) and after (gray) exposure to air for 3 h show a minor decrease in the H_2_ oxidation current (Fig. 4*A*), demonstrating that the enzyme embedded in the redox-active film remains O_2_-stable (29, 31). The catalytic current retains 74 ± 15% of its initial value even after 18 h of exposure to ambient air following an initial activation cycle under H_2_ (green in Fig. 4*B*). This stability is attributed to CbA5H’s intrinsic protection mechanism, which relies on reversible inactivation of its active site under oxidative conditions. In contrast, the sulfide-protected FeFe hydrogenase from *Desulfovibrio desulfuricans* only tolerates air exposure prior to activation. Once activated by H_2_, subsequent exposure to O_2_ leads to rapid and irreversible inactivation. After just 1 h of air exposure, the H_2_ oxidation current drops below 11%, and complete loss of activity is observed after 3 h (gray in Fig. 4*B*). The capacity of CbA5H/DV2 modified electrodes to withstand repeated activations and air exposures under conditions of open circuit were also evaluated by CV (Fig. 4*C*). The decrease in H_2_ oxidation current is about 20% per exposure, resulting in an overall loss of activity of about 65% after four cycles. The same behavior is observed for H_2_ production. This overall trend is consistent with previous findings from solution-phase assays by Rutz et al. (30), who reported a 75% loss in catalytic activity of CbA5H for H_2_ production after two exposures to air. The similar losses observed both in redox-active films for H_2_ oxidation and H_2_ evolution and in solution-phase H_2_ evolution assays suggest that the decline in activity primarily arises from enzyme inactivation rather than limitations of the immobilization matrix.

### Protection from O_2_ during H_2_ Oxidation.

The CA experiment in Fig. 4*D* shows that the H_2_ oxidation current recorded with CbA5H in PV2 under a mixed H_2_ and O_2_ gas feed is barely affected by O_2_. As observed before with the O_2_-sensitive FeFe hydrogenase from *Chlamydomonas reinhardtii*, the decrease in current that results from exposure to O_2_ is small and reversible (7). The reason is understood by examining the concentration profiles in the *Right* column of Fig. 3 and *SI Appendix*, Figs. S8 and S9). The penetration of O_2_ in the film (red curve) inactivates the enzyme molecules that are present in the outer part of the film (black concentration profile, labeled E_i_, where “i” is short for “inactive”), and oxidizes the viologen (pink), which progresses inward due to charge hopping between neighboring viologens (in an “apparent” diffusion process) (42). The oxidized viologen in turn, oxidizes the active enzyme, which oxidizes H_2_. This reaction regenerates the reduced viologen (blue), which shuttles the electrons outward via hopping and reacts with the incoming O_2_, impeding its penetration into the inner layers of the film (40).

The simulations show that the O_2_ penetration and the progress of the front of inactive enzyme is faster with DV2 than with DV1. This is because the rate of progress of the front depends on the concentration of reduced viologen (see e.g., equation 3 in ref. 35). The ratio V_ox_/V_red_ equilibrates with the H^+^/H_2_ couple in the bulk of the film. Switching from DV1 to DV2 corresponds to a decrease in *E*^0^, which leads to a decrease in the equilibrium concentration of reduced viologen, thus making the front move faster. The simulations for both DV1 and DV2 also show that catalysis for current generation is confined to the region near the electrode which has not been exposed to O_2_. This implies that the (reversible) inactivation of the enzyme in the outer layer of the film does not impact the H_2_ oxidation current and that the initial current is fully recovered when O_2_ is removed (40).

Since the protection from O_2_ provided by the redox-active film is entirely dependent on H_2_ oxidation in the outer part of the film, the enzyme should inactivate entirely if the film is exposed to O_2_ in the absence of H_2_. However, due to the intrinsic air stability of the enzyme (that results from the binding of the cysteine to the active site), the hydrogenase remains protected not only during aerobic and anaerobic H_2_ oxidation catalysis but also during storage between operational cycles and idle periods associated with intermittent use.

## Discussion

The discovery of a FeFe hydrogenase that retains H_2_-evolving activity upon repeated exposure to air came as a surprise (31), since all previously identified FeFe hydrogenases are O_2_-labile (that is, their active site is irreversibly damaged upon exposure to O_2_) (9). Compared to NiFe hydrogenases, which have been preferred for most applications so far, the advantage of using a FeFe hydrogenase is that their production can be scaled up by using an artificial maturation procedure that involves the separated large-scale productions of the apo-enzyme and its cofactor and their reaction to reconstitute the holo enzyme with full activity (18, 43).

The enzyme from *C. beijerinckii* (31) is one of only two FeFe hydrogenases identified so far that are air-stable. Happe et al. have indeed recently identified and characterized a second air-stable FeFe hydrogenase that is phylogenetically distinct from CbA5H (44). Despite their evolutionary differences, both enzymes share a common structural feature responsible for their oxygen tolerance: the coordination of a nearby cysteine side-chain to the active site iron atom (29, 44, 45).

This protection makes the enzymes easy to handle in air, but it has a downside: It also inactivates the enzyme under exposure to O_2_ or even mildly oxidizing, anaerobic conditions, so that the enzyme cannot oxidize H_2_ in air (unlike O_2_-tolerant NiFe hydrogenases) (9). Morra et al. did use CbA5H for anaerobic H_2_-driven NADPH regeneration, but the turnover frequency in the H_2_ oxidation direction was indeed very low (46). It therefore seems that because of the tradeoff between O_2_-stability and activity, the enzyme from *C. beijerinckii* cannot be very useful for H_2_ oxidation (19, 29–31). And yet, we demonstrated in this paper that this enzyme can actually be used to continuously oxidize H_2_, even in the presence of O_2_, on condition that it is embedded in a redox-active matrix with a finely tuned redox potential. The matrix prevents oxidative inactivation by maintaining the Nernst potential within a narrow range, high enough to oxidize the enzyme, which oxidizes H_2_, but below the inactivation potential of the enzyme. As shown in the *Left* column of Fig. 3, when the electrode potential is high, the viologen Nernst potential that is experienced by the enzyme matches the electrode potential in only a small region near the electrode surface, then it decreases to the standard potential of the viologen in the reaction layer, and finally to the H^+^/H_2_ equilibrium potential in the main part of the film (Fig. 3, *Left* column). The effect of the mediator is therefore to buffer the potential of the film at a value close to its standard potential. The latter can be adjusted by synthetic engineering (34). The range of useful viologen standard potentials is up to ≈ −0.3 V, at pH 7. PV2 and DV2 (*E*^0^ of −0.4 and −0.43 V vs. SHE, respectively) fall within this range and effectively preserve catalytic activity, unlike DV1, which has a significantly higher *E*^0^ leading to oxidative inactivation of the enzyme (Fig. 2*D*).

The intrinsic protection of the enzyme from O_2_, which involves a flexible cysteine residue and movement of an internal loop (29), remains functional even when the enzyme is embedded in the redox-active matrix (Fig. 4 *A*–*C*), as this conformational change is not hindered by the immobilization. This self-protection of the enzyme is maintained efficiently during long-time exposure to ambient air without the need for any treatment (no addition of extrinsic ligand or applied potential). This protection is not perfect: The delayed response to O_2_ attack (29) results in a fraction of the enzyme being irreversibly damaged after each exposure to O_2_. Therefore, repeated exposure to air gradually reduces the catalytic activity. This behavior is reminiscent of the results of solution assay experiments conducted with CbA5H, where repeated exposure to air led to near-complete activity loss after four cycles. This observation confirms that the current loss that we see in Fig. 4*C* is due to the enzyme itself, not to the entrapment of the enzyme in the redox-active film compromising its intrinsic stability. Efforts to enhance the stability of CbA5H through mutagenesis have been successful (30).

Under conditions of H_2_ oxidation catalysis in the presence of O_2_, the general mechanism for protection from O_2_ that we proposed for all hydrogenases embedded in redox-active films, including NiFe and standard FeFe hydrogenases, is also operational with CbA5H: A fraction of the incoming H_2_ is sacrificed to reduce the incoming O_2_ and prevent its penetration in the film thus protecting the enzyme within the film while a fraction of the enzyme near the film–electrolyte interface is inactivated (Fig. 4*D* and *Right* column of Fig. 3). However, with CbA5H, the inactivation of the enzyme that occurs near the film/solution interface is reversible. We have shown before, when we compared the immobilization of a NiFe hydrogenase and a more O_2_-resistant variant of the same enzyme, that this reductive reactivation makes it possible to decrease the thickness of the film, thus increasing catalyst utilization without compromising long-term stability (40).

With the mediator in the right window of potential, the enzyme CbA5H, which is a very poor H_2_ oxidation catalyst under conditions of DET, becomes very efficient in H_2_ oxidation, revealing that the active site of CbA5H is not particularly tuned to H_2_ evolution. The situation is reminiscent of two other FeFe hydrogenases from a different group (group B), the 3^rd^ hydrogenase from *Clostridium pasteurianum* (CpIII) (45, 47) and that from *Thermosediminibacter oceani* (ToHydA) (44), which inactivate under moderately oxidizing conditions as a result of the binding of a cysteine to the active site. The inactivation under oxidizing conditions makes these enzymes poor catalysts of H_2_ oxidation in solution assays (45, 47). Our work with CbA5H shows that when this inactivation is prevented, the H_2_ oxidation activity is recovered. It therefore appears that no FeFe hydrogenase identified so far is particularly tuned to be efficient in H_2_ evolution, although various oxidative inactivation mechanisms may hide that they are efficient H_2_ oxidation catalysts. This opens the perspective for a greater diversity of hydrogenases, beyond Cb5AH, which could benefit from integration into redox-active matrices to unlock their full potential for H_2_ oxidation.

## Materials and Methods

The viologen-modified dendrimers (DV1 and DV2) and polymer (PV2) were synthesized and fully characterized according to previously reported procedures (32, 34).

Preparation of the [FeFe]-hydrogenase CbA5H from **C.* beijerinckii* was carried out as previously described (20). For MET experiments, enzyme samples were exposed to air after in vitro maturation to induce H_inact_ formation. The [FeFe]-hydrogenase DdHydAB from *D. desulfuricans* was prepared according to earlier work (48) and protected against inactivation by molecular oxygen through the addition of Na_2_S under oxidative conditions (25, 34, 48).

All electrochemical measurements were carried out in a three-electrode set-up consisting of a glassy-carbon rotating disc electrode (GC-RDEs, d = 3 mm, for MET) and a rotating pyrolytic graphite edge electrode (4 × 4 mm^2^, for DET). A Pt-wire was used as a counter electrode and an Ag/AgCl 3 M KCl as a reference electrode. MET experiments were conducted in Tris/citrate buffer (50 mM) with KCl (100 mM) as the supporting electrolyte. DET experiments were conducted in phosphate buffer (100 mM) with NaCl (100 mM).

Enzyme films for DET experiments were prepared under anaerobic conditions by drop-casting 3 µL of a 10 µM solution of hydrogenase on a Pyrolytic Graphite Edge rotating disc electrode. The casted electrode was incubated for 3 min and rinsed with Milli-Q water to remove any unbound enzyme molecules.

Unless stated otherwise, the hydrogel films for MET experiments were prepared by drop-casting a dendrimer-enzyme mixture of DV1/CbA5H (2.8 µg/0.16 nmol), DV2/CbA5H (0.57 µg/0.16 nmol), PV2/CbA5H (1.13 µg/0.16 nmol), or DV2/DdHydAB_inact_ (0.28 µg/0.1 nmol) onto the GC-RDE. The films were allowed to dry for 2 h at 37 °C under an aerobic atmosphere. All CVs were recorded with an electrode rotating in a buffer predegassed with N_2_ to create O_2_-free conditions and then saturated with H_2_. The *Dd*hydAB_inact_ in the hydrogel was reactivated via CV in H_2_-saturated buffer.

To test the O_2_-stability of CbA5H and DdHydAB in the absence of H_2_ and under MET conditions, the enzymatic films were first activated via CVs under anaerobic conditions. Subsequently, the enzymatic films were exposed to oxygen (O_2_) through storage under air at 5 °C. After exposure time, the residual activity was examined via CVs under anaerobic conditions. The procedure was repeated to determine the stability under repeated O_2_ exposure. The O_2_-stability of CbA5H during catalytic H_2_ oxidation was investigated via CA with O_2_ added at 5% into the H_2_ gas feed.

## Supplementary Material

Appendix 01 (PDF)

## Data Availability

Source data have been deposited in Zenodo (DOI: 10.5281/zenodo.15965654) (49). All other data are included in the article and/or *SI Appendix*.
