# Supplementary material for: Turning the FeFe hydrogenase from Clostridium beijerinckii into an efficient H2 oxidation catalyst using a redox-active matrix
Source: Proc Natl Acad Sci U S A. 2025 Oct 9;122(41):e2514698122. doi: 10.1073/pnas.2514698122 (PMC12541403; doi:10.1073/pnas.2514698122)
Supplement: Supplementary file 1 — Appendix 01 (PDF) [file pnas.2514698122.sapp.pdf]

PNAS

**Supporting Information for**

# Turning the FeFe Hydrogenase from *Clostridium beijerinckii* into an Efficient $H_2$ Oxidation Catalyst Using a Redox-active Matrix

Dawit T. Filmon, Jan Jaenecke, Martin Winkler, Vincent Fourmond, Christophe Léger, Nicolas Plumeré

Correspondence to Christophe Léger, Nicolas Plumeré
Email: leger@imm.cnrs.fr, nicolas.plumere@tum.de

**This PDF file includes:**

1. Supplementary figures 1 to 11
2. Supplementary text.

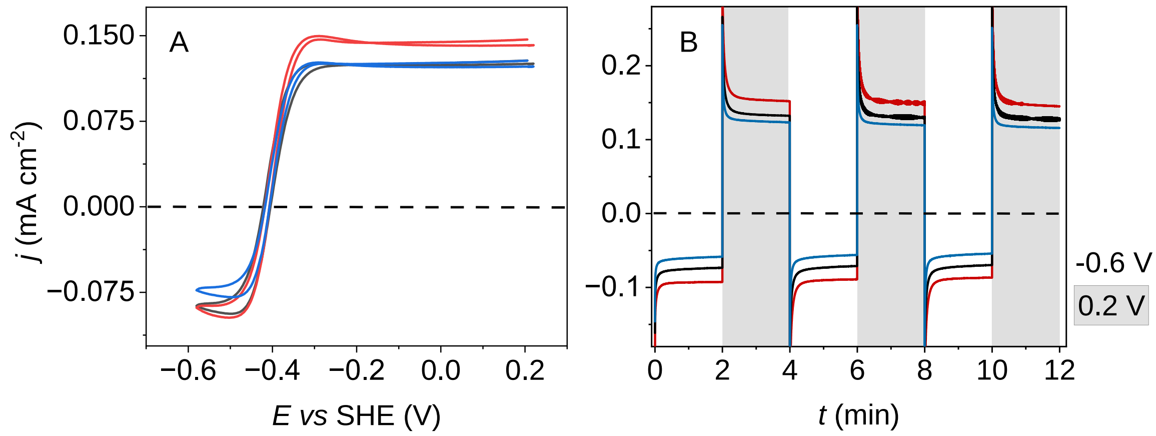


**Fig. S1. Electrochemistry of CbA5H under MET conditions**. (A) Individual CVs of CbA5H (0.04 nmol) embedded in DV2 (0.14 µg) in Tris-citrate/HCl buffer (50 mM) at pH 7.0 under 100% $H_2$. (B) Individual CAs of CbA5H at a constant applied potential of +0.2 V (gray shaded) and -0.6 V *vs* SHE (not shaded). All experiments were conducted on a rotating glassy carbon electrode (*d* = 3 mm), v = 10 mV $s^{-1}$, T = 20 °C, ω = 2000 rpm.

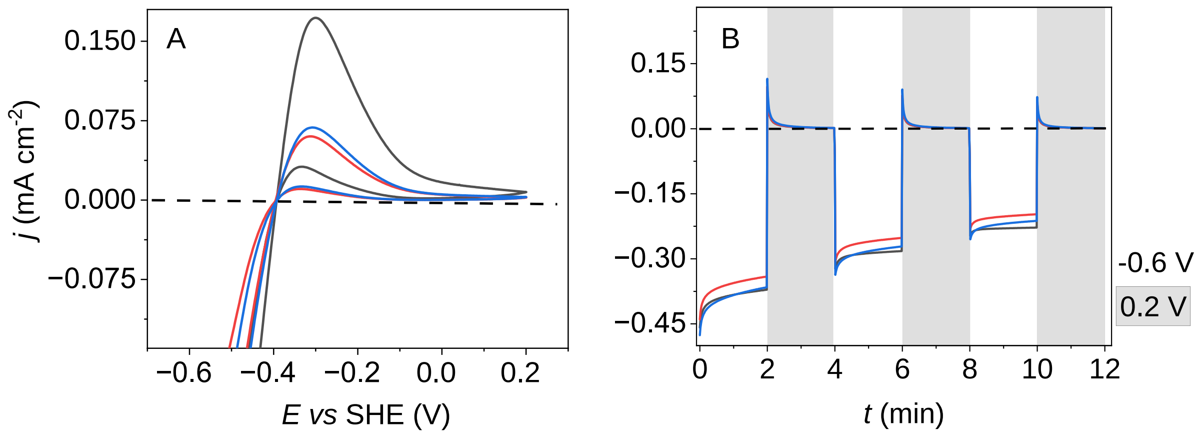


**Fig. S2**. **Electrochemistry of CbA5H under DET conditions.** (A) Individual CVs of CbA5H in Phosphate buffer (100 mM) with NaCl (100 mM) at pH 7.0 under 100% $H_2$ under DET conditions. (B) Individual CAs of CbA5H at a constant applied potential of +0.2 V (gray shaded) and -0.6 V *vs* SHE (not shaded). All experiments were conducted on a rotating pyrolytic graphite edge electrode (4×4 $mm^2$), v = 10 mV $s^{-1}$, T = 20 °C, ω = 2000 rpm.

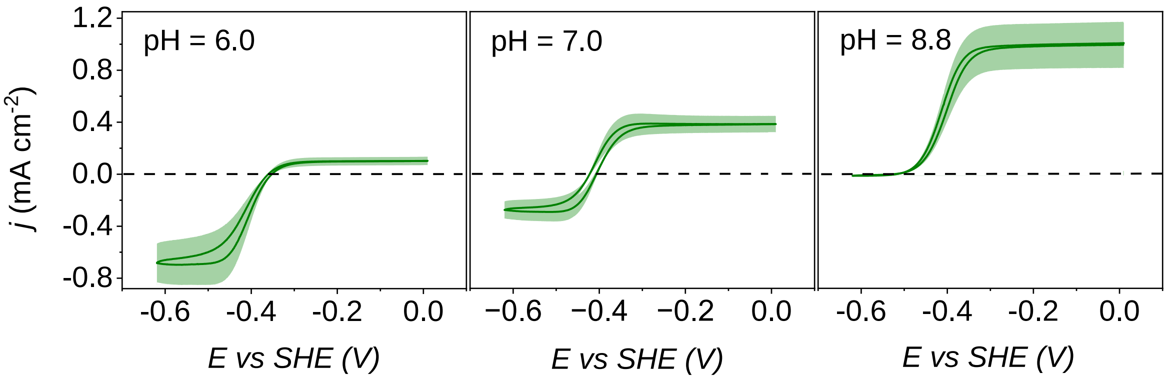


**Fig. S3**. **Catalytic bias of CbA5H embedded in DV2.** CVs of CbA5H (0.16 nmol) in DV2 (0.57 µg) in Tris-citrate/HCl buffer (50 mM) at pH 6.0, 7.0 and 8.8 (left to right) under 100% $H_2$. Three individual films were measured with the standard deviation indicated by the shaded area (n = 3). All experiments were conducted on a rotating glassy carbon electrode (*d* = 3 mm), v = 10 mV $s^{-1}$, T = 20 °C, ω = 2000 rpm.

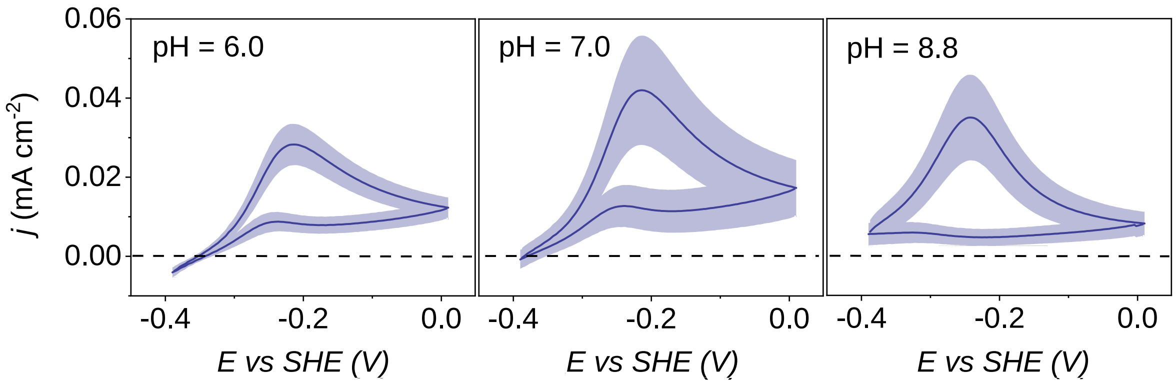


**Fig. S4. Catalytic bias of CbA5H embedded in DV1.** CVs of CbA5H (0.16 nmol) in DV1 (2.8 µg) in Tris-citrate/HCl buffer (50 mM) at pH 6.0, 7.0 and 8.8 (left to right) under 100% $H_2$. Three individual films were measured with the standard deviation indicated by the shaded area (n = 3). All experiments were conducted on a rotating glassy carbon electrode (*d* = 3 mm), v = 10 mV $s^{-1}$, T = 20 °C, ω = 2000 rpm.

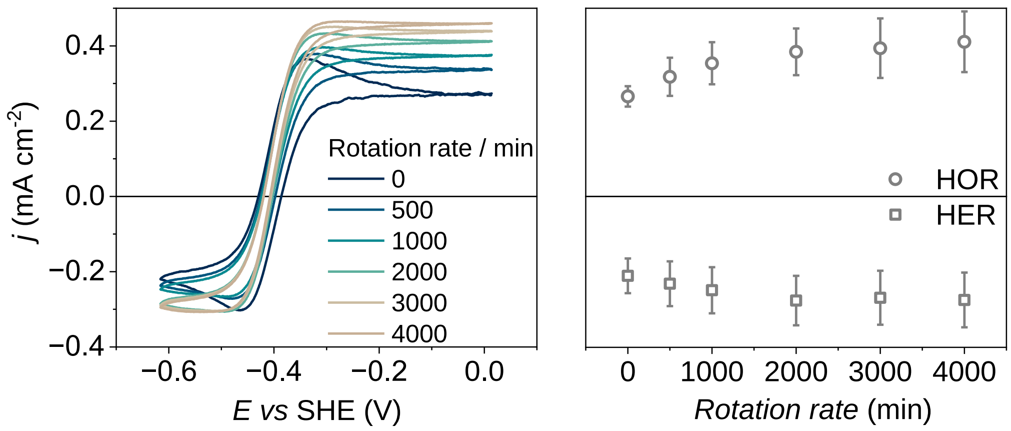


**Fig. S5. Effect of the rotation rate on catalytic current density of CbA5H embedded in DV2.** (A) CVs of CbA5H (0.16 nmol) in DV2 (0.57 µg) in Tris-citrate/HCl buffer (50 mM) at pH 7.0 and different electrode rotation rates. (B) Plot of steady-state current densities *vs* the rotation rate of the electrode. The catalytic current densities are independent of the rotation rate at high rotation rates, which excludes the depletion of $H_2$ in the redox-active film. Three individual films were measured with the standard deviation indicated by the error bars (n = 3). All experiments were conducted on a rotating glassy carbon electrode (*d* = 3 mm), v = 10 mV $s^{-1}$, T = 20 °C.

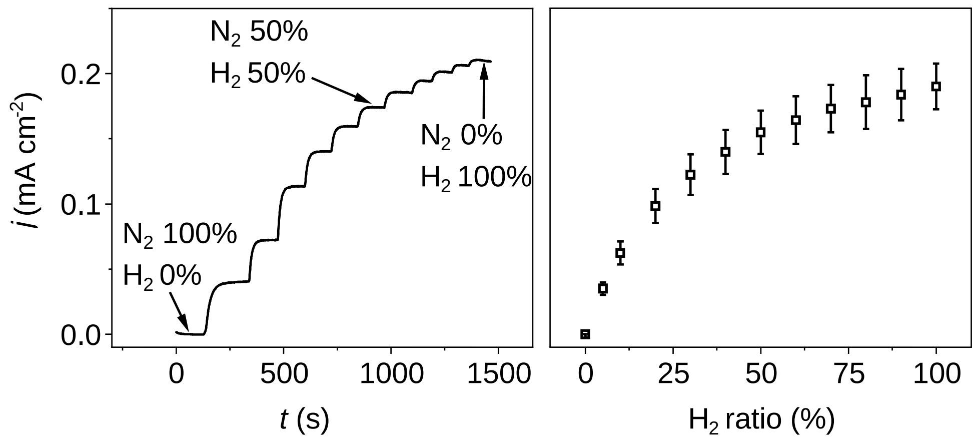


**Fig. S6. Effect of the $H_2$ partial pressures on catalytic current density of CbA5H embedded in DV2.** (A) CA of CbA5H (0.08 nmol) in DV2 (0.28 µg) at pH 7.0 in Tris-HCl/Citrate (50 mM) with KCl (100 mM) at -0.2 V *vs* SHE. The gas feed mixture of $N_2$:$H_2$ varied as indicated. (B) Plot of catalytic current density *vs* the $H_2$ partial pressure. At high concentrations of $H_2$, the catalytic current is independent of $H_2$ concentration. Three individual films were measured with the standard deviation indicated by the error bars (n = 3). All experiments were conducted on a rotating glassy carbon electrode ($d$ = 3 mm), v = 10 mV $s^{-1}$, T = 20 °C, ω = 2000 rpm.

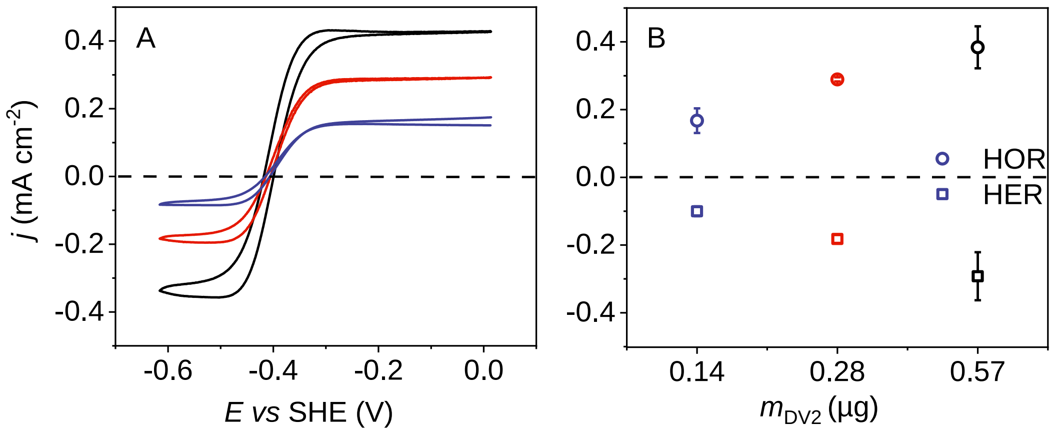


**Fig. S7. Effect of the film thickness on catalytic current density of CbA5H embedded in DV2.** (A) CVs of CbA5H embedded in DV2 films in Tris-citrate/HCl buffer (50 mM) with KCl (100 mM) at pH 7.0. The films were prepared by dropcasting different amounts of a mixture of CbA5H/DV2 (0.16 nmol/0.57 µg (black), 0.08 nmol/0.28 µg (red) and 0.04 nmol/0.14 µg (purple), keeping the ratio of enzyme to dendrimer constant. (B) Plot of steady-state current densities for catalytic current for the HOR (open circle) and HER (open squares) *vs* the amount of DV2 loading. Three individual films were measured with the standard deviation indicated by the error bars (n = 3). With increasing loading of CbA5H/DV2, a linear increase in catalytic current densities can be observed without any visible enzyme inactivation at high potentials. This confirms that the enzyme inactivation at oxidative potentials is achieved independently of the film thickness. All experiments were conducted on a rotating glassy carbon electrode (*d* = 3 mm), v = 10 mV $s^{-1}$, T = 20 °C, ω = 2000 rpm.

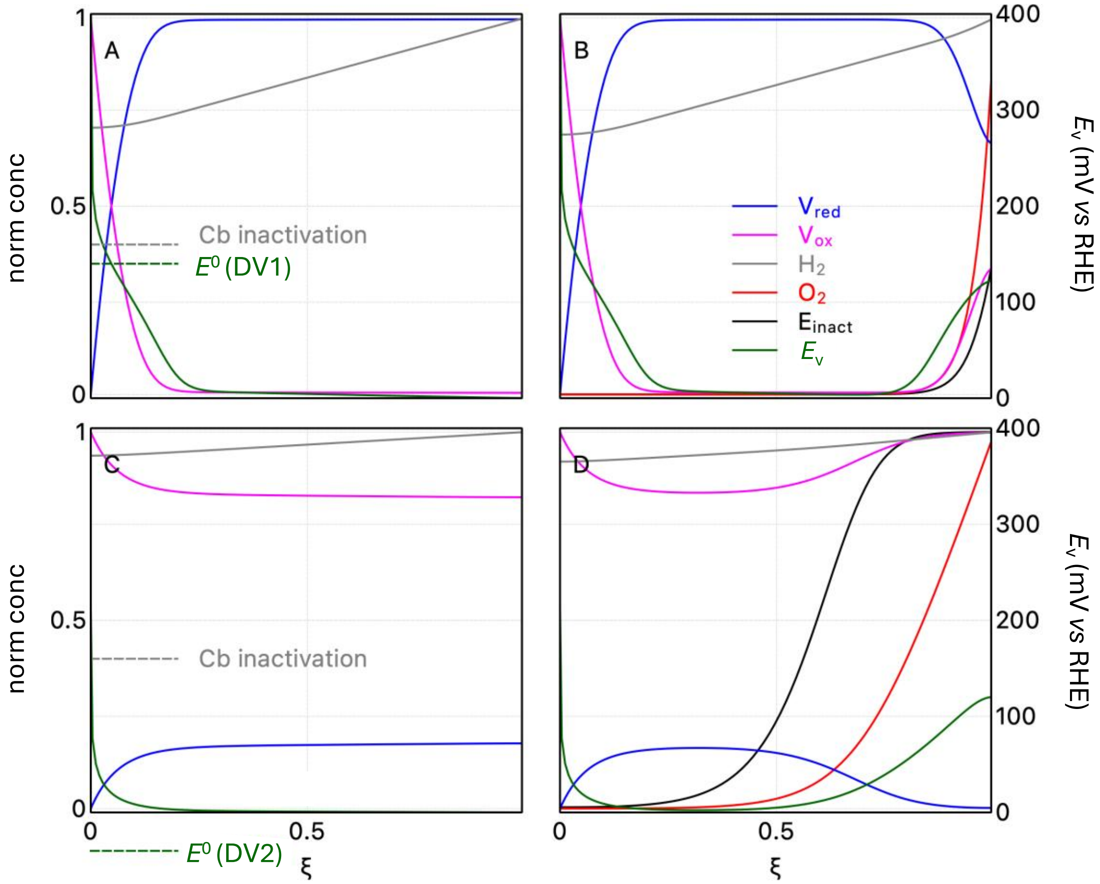


**Fig. S8. Simulated concentration profiles of enzymatic films at pH 6.0.** The results of simulations showing the distribution of the different species within the film at pH 6.0. The figures show the concentration profiles of $V_{ox}$ (pink), $V_{red}$ (blue), $H_2$ (gray), $O_2$ (red), and inactive enzyme (black), and the viologen Nernst potential vs RHE (green) (calculated using the Nernst equation from the ratio $V_{ox}/V_{red}$) as a function of the normalized distance from the electrode, calculated under oxidizing, anaerobic conditions (left, panels A, C) and aerobic conditions (right, panels B, D), with viologen potentials matching those of DV1 (top, panels A, B) and DV2 (bottom, panels C, D). The standard potentials of the viologens and the potential where the enzyme inactivates are shown as horizontal dashed lines.

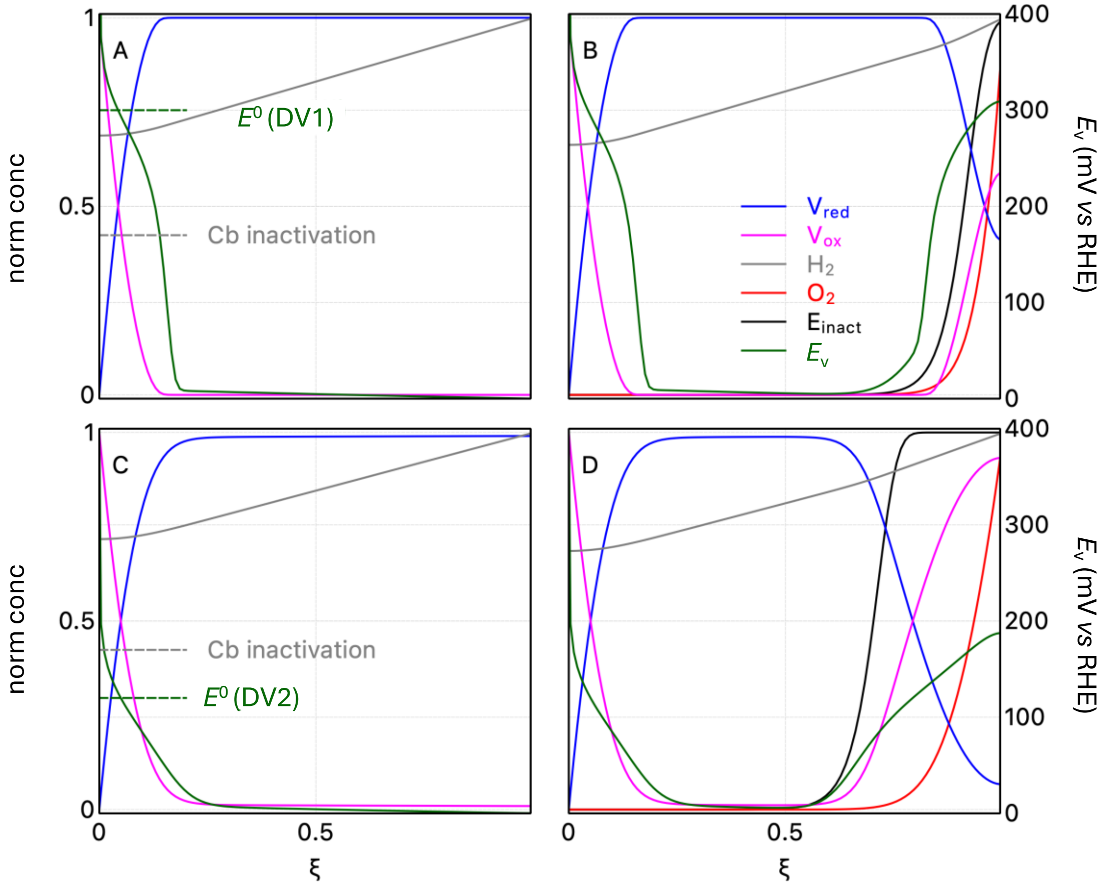


**Fig. S9. Simulated concentration profiles of enzymatic films at pH 8.8.** The results of simulations showing the distribution of the different species within the film at pH 8.8. The figures show the concentration profiles of $V_{ox}$ (pink), $V_{red}$ (blue), $H_2$ (gray), $O_2$ (red), and inactive enzyme (black), and the viologen Nernst potential vs RHE (green) (calculated using the Nernst equation from the ratio $V_{ox}/V_{red}$) as a function of the normalized distance from the electrode, calculated under oxidizing, anaerobic conditions (left, panels A, C) and aerobic conditions (right, panels B, D), with viologen potentials matching those of DV1 (top, panels A, B) and DV2 (bottom, panels C, D). The standard potentials of the viologens and the potential where the enzyme inactivates are shown as horizontal dashed lines.

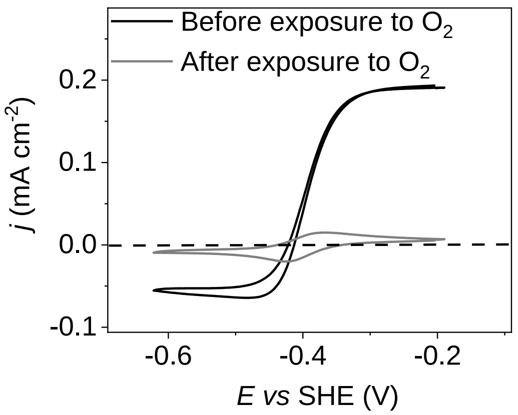


**Fig. S10. $O_2$ stability of *Dd*HydAB embedded in DV2.** CVs of DdHydAB (0.1 nmol) in DV2 (0.28 µg) in Tris-citrate/HCl buffer (50 mM) at 7.4 and under 100% $H_2$ before (purple) and after (gray) 3 hours of exposure to air. The shape of the CV changes from a sigmoidal shape to a diffusion-limited CV, confirming the loss of catalytic activity due to $O_2$-based enzyme inactivation. All experiments were conducted on a rotating glassy carbon electrode (*d* = 3 mm), v = 10 mV $s^{-1}$, T = 20 °C, ω = 2000 rpm.

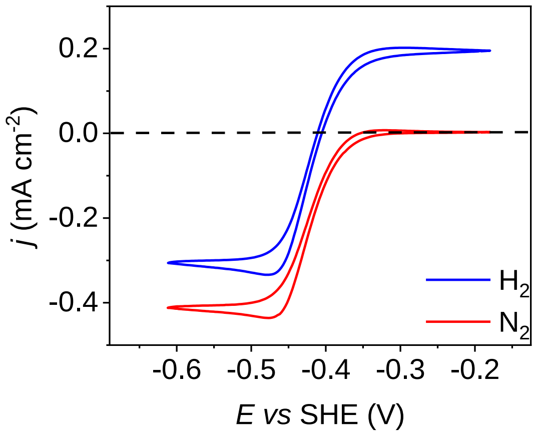


**Fig. S11. Electrochemistry of CbA5H under MET conditions embedded in a PV2.** CVs of CbA5H (0.16 nmol) in PV2 (1.13 µg) in Tris-HCl/Citrate (50 mM) with KCl (100 mM) at pH 7.4 under 100% $H_2$ (blue) and 100% $N_2$ (red). All experiments were conducted on a rotating glassy carbon electrode (*d* = 3 mm), v = 10 mV $s^{-1}$, T = 20 °C, ω = 2000 rpm.

# SI - 2 : The Model of Bidirectional Enzyme Catalysis Under Conditions of MET in a Film

## 1 The assumptions

The simulations shown in main text fig. 3 are based on the model described below, which extends our previous work[1–3] by considering that the catalyzed reaction is reversible (bidirectionnal). The terms added compared to our previous work are shown in red.

The model considers

- the conversion between oxidized (A) and reduced (B) viologen at the electrode (eq 1a). This interfacial electron transfer is supposed to be fast and to remain at equilibrium.
- The oxidation of the reduced enzyme ($E_2$ to $E_1$) by A with 2nd order rate constant $k_a$, and the reverse reaction with rate constant $k_b$ (eq 1b).
- The oxidation of the substrate hydrogen ("S") by the oxidized enzyme with a 2nd order rate constant $k_{\text{cat}}/(K_m + S)$ (we note $S$ the concentration of S), and the reverse reaction with pseudo-first order rate constant $k_p\,K_m/(K_m + S)$ (eq 1c).

In the presence of oxygen, and as in our previous work, we also consider

- The bimolecular reaction of oxygen with the reduced or the oxidized enzyme to produce an inactive form of the enzyme $E_1$ (eqs 1d and 1e).
- The oxidation of the reduced viologen B by oxygen (eq 1f).

We neglect the reactivation of the inactive enzyme by reduced viologen.

$$\mathrm{A} + \mathrm{e}^- \underset{}{\overset{\text{fast}}{\rightleftharpoons}} \mathrm{B} \tag{1a}$$

$$\mathrm{E_2} + \mathrm{A} \underset{k_b}{\overset{k_a}{\rightleftharpoons}} \mathrm{E_1} + \mathrm{B} \tag{1b}$$

$$\mathrm{E_1} + \mathrm{S} \underset{k_p K_m/(K_m+S)}{\overset{k_{\text{cat}}/(K_m+S)}{\rightleftharpoons}} \mathrm{E_2} \tag{1c}$$

$$\mathrm{E_1} + \mathrm{O} \xrightarrow{k_i} \mathrm{E_i} \tag{1d}$$

$$\mathrm{E_2} + \mathrm{O} \xrightarrow{k_i} \mathrm{E_i} \tag{1e}$$

$$\mathrm{B} + \mathrm{O} \xrightarrow{k_o} \mathrm{A} \tag{1f}$$

The equilibrium constant for equation (1c) is:

$$K_1 = \frac{E_2}{E_1 \times S} = \frac{k_{\text{cat}}/(K_m + S)}{k_p K_m/(K_m + S)} = \frac{k_{\text{cat}}}{k_p K_m} \tag{2}$$

The one for equation (1b) is:

$$K_2 = \frac{E_1 \times B}{E_2 \times A} = \frac{k_a}{k_b} \tag{3}$$

The difference in potential between the mediator and the $H^+/H_2$ couples sets the product $K_1 \times K_2$. The sum of (1b) and (1c) gives:

$$\mathrm{A} + \mathrm{S} \rightleftharpoons \mathrm{B} \tag{4}$$

The equilibrium constant is:

$$\frac{B}{A \times S} = \frac{B \times E_2 \times E_1}{A \times S \times E_1 \times E_2} = K_2 \times K_1 \tag{5}$$

If one calls

$$\Delta E = E^0_{A/B} - E'^0_{\mathrm{H}^+/\mathrm{H}_2} \tag{6}$$

in which $E'^0_{\mathrm{H}^+/\mathrm{H}_2}$ is the apparent potential at the given pH, then

$$\frac{B \times S_0}{A \times S} = \exp\frac{-F\Delta E}{RT} = K_1 K_2 S_0 \tag{7}$$

where $S_0$ corresponds to the "standard solubility" of $H_2$, that is the concentration of $H_2$ in equilibrium with 1 bar of $H_2$.

As a consequence,

$$\frac{k_{\mathrm{cat}}\, S_0}{k_p K_m} \times \frac{k_a}{k_b} = \exp\frac{-F\Delta E}{RT} \tag{8}$$

## 2 The initial set of differential equations

According to the above set of chemical equations and definition of the rate constants, the concentrations evolve according to:

$$\frac{\partial E_1}{\partial t} = -\frac{k_{\mathrm{cat}} S E_1 - k_p K_m E_2}{K_m + S} + k_a A E_2 - k_b E_1 B - k_i E_1 O \tag{9}$$

$$\frac{\partial E_i}{\partial t} = k_i (E_1 + E_2) O \tag{10}$$

$$\frac{\partial A}{\partial t} = D_A \frac{\partial^2 A}{\partial x^2} - k_a A E_2 + k_b E_1 B + k_o B O \tag{11}$$

$$\frac{\partial O}{\partial t} = D_O \frac{\partial^2 O}{\partial x^2} - k_i (E_1 + E_2) O - k_o B O \tag{12}$$

$$\frac{\partial S}{\partial t} = D_S \frac{\partial^2 S}{\partial x^2} - \frac{k_{\mathrm{cat}} S E_1 - k_p K_m E_2}{K_m + S} \tag{13}$$

## 3 The dimensionless set of differential equations

We normalized the concentrations by defining $a = A/A^\Sigma$, $b = B/A^\Sigma$, $e_i = E_i/A^\Sigma$, $o = O/A^\Sigma$, $s = S/A^\Sigma$, $\xi = x/\ell$, $\tau = t \times k_a E^\Sigma$, where $A^\Sigma$ is the total concentration of viologen in the film (oxidized plus reduced), $\ell$ is the thickness of the film and $E^\Sigma$ is the concentration of enzymes in the film.

We defined the following nondimensional parameters (most of which have already been introduced in our previous work[1–3]):

| | |
|---|---|
| $\kappa$ | $\ell\sqrt{k_a E^\Sigma / D_A}$ |
| $\mu$ | $A^\Sigma / K_m$ |
| $\kappa_i$ | $k_i A^\Sigma / k_a E^\Sigma$ |
| $\kappa_o$ | $k_o A^\Sigma / k_a E^\Sigma$ |
| $\epsilon$ | $E^\Sigma / A^\Sigma$ |
| $\delta^S$ | $D_S / D_A$ |
| $\delta^O$ | $D_O / D_A$ |
| $\alpha$ | $k_a K_m / k_{\mathrm{cat}}$ |
| $o^\infty$ | $S^\infty / A^\Sigma$ |
| $s^\infty$ | $S^\infty / A^\Sigma$ |
| $\kappa_p$ | $k_p / k_{cat}$ |
| $\kappa_b$ | $k_b / k_a$ |

Equation (8) becomes:

$$\frac{1}{\kappa_p \kappa_b} \times \frac{S_0}{K_m} = \exp \frac{-F\Delta E}{RT} \tag{14}$$

The set of non-dimensional differential equations is

$$\frac{\partial e_1}{\partial \tau} = -\frac{e_1 s - \kappa_p e_2/\mu}{\epsilon\alpha(1+\mu s)} + a\frac{e_2}{\epsilon} - \left(o\kappa_i + \kappa_b \frac{b}{\epsilon}\right) e_1 \tag{15}$$

$$\frac{\partial e_i}{\partial \tau} = \kappa_i (e_1 + e_2) o \tag{16}$$

$$\frac{\partial a}{\partial \tau} = \frac{1}{\kappa^2}\frac{\partial^2 a}{\partial \xi^2} - \frac{a}{\epsilon} e_2 + \kappa_b e_1 \frac{b}{\epsilon} + bo\kappa_o \tag{17}$$

$$\frac{\partial o}{\partial \tau} = \frac{\delta^O}{\kappa^2}\frac{\partial^2 o}{\partial \xi^2} - (e_1 + e_2) o\kappa_i - \kappa_o bo \tag{18}$$

$$\frac{\partial s}{\partial \tau} = \frac{\delta^S}{\kappa^2}\frac{\partial^2 s}{\partial \xi^2} - \frac{e_1 s - \kappa_p e_2/\mu}{\epsilon\alpha(1+\mu s)} \tag{19}$$

## 4 Steady-state hypothesis for $e_1$ and $e_2$

Since the enzymatic reaction are fast on the time scale of the evolution of the system, steady-state can be assumed for $E_1$ and $E_2$:

$$-\frac{e_1 s - \kappa_p e_2/\mu}{\epsilon\alpha(1+\mu s)} + a\frac{e_2}{\epsilon} - \kappa_b \frac{b}{\epsilon} e_1 = 0 \tag{20}$$

Noting $e_a = e_1 + e_2$ the nondimensional concentration of active enzyme, inversion gives:

$$e_1 = \frac{\mu\alpha(1+\mu s)a + \kappa_p}{\mu\alpha(1+\mu s)(a + \kappa_b b) + \kappa_p + \mu s} \times e_a \tag{21}$$

$$e_2 = \frac{\mu\alpha(1+\mu s)\kappa_b b + \mu s}{\mu\alpha(1+\mu s)(a + \kappa_b b) + \kappa_p + \mu s} \times e_a \tag{22}$$

Injecting back into the differential equations above yields:

$$\frac{\partial a}{\partial \tau} = \frac{1}{\kappa^2}\frac{\partial^2 a}{\partial \xi^2} - e_a \frac{\mu a s - \kappa_b \kappa_p (1-a)}{\epsilon[\mu\alpha(1+\mu s)(a+\kappa_b(1-a))+\kappa_p+\mu s]} + b o \kappa_o \tag{23}$$

$$\frac{\partial s}{\partial \tau} = \frac{\delta^S}{\kappa^2}\frac{\partial^2 s}{\partial \xi^2} - e_a \frac{\mu a s - \kappa_b \kappa_p (1-a)}{\epsilon[\mu\alpha(1+\mu s)(a+\kappa_b b)+\kappa_p+\mu s]} \tag{24}$$

$$\frac{\partial o}{\partial \tau} = \frac{\delta^O}{\kappa^2}\frac{\partial^2 o}{\partial \xi^2} - e_a o \kappa_i - \kappa_o (1-a) o \tag{25}$$

$$\frac{\partial e_a}{\partial \tau} = -\kappa_i e_a o \tag{26}$$

The above differential equations correspond to the following set of reactions:

$$\mathrm{e_a} + \mathrm{o} \xrightarrow{\kappa_i} \mathrm{e_i} \tag{27a}$$

$$\mathrm{e_i} + \mathrm{b} \xrightarrow{\kappa_r} \mathrm{e_a} + \mathrm{a} \tag{27b}$$

$$\mathrm{b} + \mathrm{o} \xrightarrow{\kappa_o} \mathrm{a} \tag{27c}$$

$$\mathrm{a} + \mathrm{s} \underset{k_b^{\mathrm{eff}}}{\overset{k_{as}^{\mathrm{eff}}}{\rightleftharpoons}} \mathrm{b} \tag{27d}$$

with the effective rate constants given by:

$$k_{as}^{\mathrm{eff}} = \frac{\mu e_a}{\epsilon[\mu\alpha(1+\mu s)(a+\kappa_b b)+\kappa_p+\mu s]} \tag{28}$$

$$k_b^{\mathrm{eff}} = \frac{\kappa_b \kappa_p e_a}{\epsilon[\mu\alpha(1+\mu s)(a+\kappa_b b)+\kappa_p+\mu s]} \tag{29}$$

## 5 Parameters used to run the simulations

Our goal here is not to precisely define the exact parameters, but only to use simulations to understand qualitatively the profile of potential of the viologen in the film. Therefore, we run the simulations using reasonable values of the parameters, mostly similar to those determined in our previous work,[1–3] and listed below.

We run the simulations for a film of thickness $\ell = 5\mu$m. The diffusion coefficients were 4 $10^{-5}$ cm$^2$/s for $H_2$, $10^{-5}$ cm$^2$/s for $O_2$, and $10^{-8}$ cm$^2$/s for electron hopping. We set the Michaelis constant for $H_2$ to a typical value for FeFe hydrogenases,[4] $K_M = 1$ mM. The total concentration of viologen in the film was also typical of this design, $A^\Sigma = 0.1$ M. The rate constants $k_{\mathrm{cat}}$ and $k_p$ were 3000 s$^{-1}$, and 3000 s$^{-1}$, respectively, to account for the nearly equal activity of the enzyme in both directions. The product $k_a k_b$ was kept constant, equal to (5 $10^4$ M$^{-1}$s$^{-1}$)$^2$, and the ratio $k_a/k_b$ was deduced from eq 8, for a chosen value of $\Delta E$, and $K_M = S^\infty = S_0 = 1$ mM (the concentration of $H_2$ equilibrated with 1 atm. of $H_2$). The values of $k_a$ and $k_b$ were then deduced from the product and the ratio of $k_a$ and $k_b$. The rate constant of viologen oxidation by $O_2$ was $k_o = 0.3\ 10^6$ M$^{-1}$s$^{-1}$, the rate of inactivation of the enzyme by $O_2$ was typical for FeFe hydrogenases,[5] $k_i = 10^3$ M$^{-1}$s$^{-1}$. The bulk concentration of $O_2$ was 0.05 mM (corresponding to about 5% $O_2$).

In addition to the simulations shown in main text Fig. 3, the results of the same calculations at pH 6 and 8.8 are shown in SI-Fig. 8 and SI-Fig. 9, respectively.

# References


[1] Li, H.; Buesen, D.; Dementin, S.; Léger, C.; Fourmond, V.; Plumeré, N. *J. Am. Chem. Soc.* **2019**, *141*, 16734–16742.

[2] Fourmond, V.; Stapf, S.; Li, H.; Buesen, D.; Birrell, J.; Rüdiger, O.; Lubitz, W.; Schuhmann, W.; Plumeré, N.; Léger, C. *J. Am. Chem. Soc.* **2015**, *137*, 5494–5505.

[3] Fourmond, V.; Léger, C. *ChemElectroChem* **2021**, *8*, 2607–2615.

[4] Fourmond, V.; Baffert, C.; Sybirna, K.; Dementin, S.; Abou-Hamdan, A.; Meynial-Salles, I.; Soucaille, P.; Bottin, H.; Léger, C. *Chem. Commun.* **2013**, *49*, 6840–6842.

[5] Kubas, A.; Orain, C.; De Sancho, D.; Saujet, L.; Sensi, M.; Gauquelin, C.; Meynial-Salles, I.; Soucaille, P.; Bottin, H.; Baffert, C.; Fourmond, V.; Best, R. B.; Blumberger, J.; Léger, C. *Nat. Chem.* **2017**, *9*.
